# Integrated Care for Older Adults: A Struggle for Sustained Implementation in Northern Netherlands

**DOI:** 10.5334/ijic.5434

**Published:** 2020-07-13

**Authors:** Sander Holterman, Maarten Lahr, Klaske Wynia, Marike Hettinga, Erik Buskens

**Affiliations:** 1 University Medical Centre Groningen, Department of Epidemiology, Unit Health Technology Assessment, Groningen, NL; 2 Windesheim University of Applied Sciences, Research Group IT Innovations in Healthcare, Zwolle, NL; 3 University of Groningen, University Medical Centre Groningen, Department of Health Sciences, Community and Occupational Medicine, Groningen, NL; 4 University of Groningen, University Medical Centre Groningen, Department of Neurology, Groningen, NL; 5 University of Groningen, Department of Operations, Faculty of Economics and Business, Groningen, NL

**Keywords:** integrated care, implementation, sustainability, payment models, transformation

## Abstract

**Introduction:**

Integrated care has been suggested as a promising solution to the disparities in access and sustained high quality long-term care emerging in Europe’s ageing population. We aim to gain a better understanding of context-specific barriers to and facilitators of implementation of integrated care by doing a retrospective assessment of seven years of Embrace. This Dutch integrated person-centred health service for older adults was based on two evidence-based models (the Chronic Care Model and the Kaiser Permanente Triangle). Despite successful deployment the programme ended in 2018. In this case study we assess the impact of the programme based on past evaluations, reflect on why it ended, lessons learned and ideas to take forward.

**Discussion:**

The majority of health outcomes were positive and the perceived quality of care improved, albeit no clear-cut savings were observed, and the costs were not balanced across stakeholders. The Embrace payment model did not support the integration of health services, despite reforms in long-term care in 2015.

**Key lessons:**

Enabling policy and funding are crucial to the sustained implementation of integrated person-centred health services. The payment model should incentivize the integration of care before the necessary changes can be made at organizational and clinical levels towards providing proactive and preventive health services.

## Introduction

Throughout Europe, communities and entire societies have to face the challenges that come with ageing populations. As the number of people with chronic diseases and multi-morbidity increases, the need for long-term care will rise to a point where budgets become limitative and disparities in access to high-quality care will occur. This is considered a growing public health concern [1].

According to the World Health Organization (WHO), person centred integrated care services provide promising solutions to these challenges since they have the potential to enhance the quality of and access to healthcare, thus improving clinical outcomes and patient satisfaction while reducing cost [2]. These potential outcomes is in line with the three dimensions that Berwick et al. (2008) defined as the Triple Aim: 1) improving the patient experience of care, 2) improving the health of populations, and 3) reducing the per capita cost of healthcare [3]. Importantly, such an integrated strategy for person-oriented health services has fundamental consequences for organizational and financial structures [2,4].

Although systematic literature reviews have identified numerous elements, factors, barriers to and facilitators of successful implementation at the micro, meso and macro level [5,6,7], international experience of integrated care models that provides evidence on their impact is limited [8]. The evidence available for strategies that support integration focus primarily on the operational processes (i.e. micro level) and are often studied in a specific setting and for a specific target group [7]. Rutten-van Mölken (2017) concluded that a better understanding of the context of best practices in integrated care is needed to distinguish between generic and context-specific barriers to and facilitators of implementation [9]. Elements of integrated care at the policy and system level (i.e. meso and micro level), such as financial incentives and payment schemes, are too often marginalized [10].

To assess and address the challenges of implementing integrated care in the context of the Dutch healthcare system, we will examine Embrace, a combination of the evidence-based Chronic Care Model (CCM) [11] and a population health management model (the Kaiser Permanente Triangle [12]), transferred to the Dutch healthcare system and specified for older adults (aged 75 years and older). Embrace focuses on the wellbeing of older adults and aims to provide support to enable older adults to live at home for as long as is possible and desirable. This is generally desired, provided that the same or a better quality of care is achieved at a lower cost compared to residential care. Indeed, although the programme had a positive impact overall on perceived quality of care, health outcomes, per capita cost and care utilization, sustained implementation could not be achieved.

We have examined the integrated care programme Embrace, from its start in 2012 until it ended in 2018. In this paper, we describe the initial goals and contextual changes, and summarise the impact of the programme based on past evaluations. In addition, we reflect on why the programme ended and the legacy it left behind. Finally, we present lessons learned and conclude with prospects for the revival of the programme.

## Methods

### Study design

We used a mixed-methods case study design [13] to retrospectively assess the implementation and discontinuation of Embrace within the Dutch context.

### Data collection

Data for this case study were collected in multiple ways, as summarized in Table 1. For information on the context of the Dutch healthcare system, the 2015 International Profiles of Health Care Systems report [14] and journal articles on the long-term care reform in 2015 [15,16] were used. To describe the key elements and outcomes of Embrace, information from journal articles, project reports, intervention protocols and financial agreements was collected.

**Table 1:** Summary of data collection.

Data collection method	Used for information on

1. Documents: programme reports and journal articles	Context, intervention, payment model, impact
2. PDSA-cycle documents	Barriers and facilitators
3. Semi-structured interviews	Intervention, payment model, barriers and facilitators

Semi-structured interviews were conducted with three Embrace programme coordinators to gain a deeper understanding of three topics: (1) integrated care characteristics, (2) evaluation and implementation and (3) sustainability and the payment model. The same researcher (SH) conducted the interviews, which lasted approximately 90 minutes, using an interview guide. The interviews were audio-recorded and transcribed afterwards.

Part of the data collected originated from the ACT@Scale project (2016–2019, EU Grant Agreement 709770) in which Embrace participated as a case study of scaling up integrated care [17]. This ACT@Scale documentation was used to identify the barriers to and facilitators of the implementation and scaling process.

### Data analysis

Thematic analyses (type: template analysis) [18] was used on a pragmatic analysis of the Plan-Do-Study-Act-documents and the interview transcripts. These were coded and categorised manually, i.e. not using a specific coding tool, and Microsoft Excel was used to support the thematic analysis. We structured this information based on the three levels and the underlying components of the scoping review by Threapleton et al. (2017) [6]. For the emerging barriers and facilitators, the first author (SH) did the analysis and discussed it with two co-authors (ML, EB). The semi-structured interviews with the programme coordinators were analysed by the first author (SH) and discussed it with one co-author (ML). Relevant documents on Embrace (i.e. agreements, articles, reports, evaluations and agreements) were used to 1) reconstruct the process from the first implementation until the programme ended in 2018, 2) describe the payment model and 3) summarise the impact. We looked for information on critical decisions and the reasons why they were made, but also to clarify or contextualize information obtained from the interviews, by triangulation.

### Ethics statement

This study did not require the participation of human subjects, nor actual patient data. The information collected with the semi-structured interviews was anonymous and non-identifiable information or data related to individuals were collected or accessible. As such, this retrospective assessment was considered “service evaluation” and therefore beyond the scope of the local UMCG Research Ethics Committee. The latter only applies to experimental studies on human subjects.

## The intervention

Embrace has previously been described in great detail [19,20]. In brief, Embrace provided person-centred, integrated, proactive and preventive care and support reflecting the key elements of the Chronic Care Model (CCM)[11]: self-management support, delivery system design, decision support and clinical information systems. Community-living adults aged 75 years and older were classified in terms of risk profiles (Robust, Frail and Complex Care Needs) based on the self-reported complexity of care needs and the level of frailty. The care and support deemed appropriate to each risk profile was then provided by an Elderly Care Team comprising a general practitioner (GP), an elderly care physician, a community nurse and a social worker. The GP led the team and was accountable. The characteristics of the intervention per risk profile are summarized as Supplementary Material, Table 1.

The clinical information system consisted of a Digital Elderly Record that was accessible for the members of the Elderly Care Team. It included personal data, the results of the questionnaires, the anamnesis, the advanced care plan and information on issues to be discussed in the Elderly Care Team meetings. The providers’ staff received intensive training in using the protocol and the clinical information system for over seven days in total. They were coached during the Elderly Care Team meetings for over two years and received peer-to-peer support.

**Figure 1:**
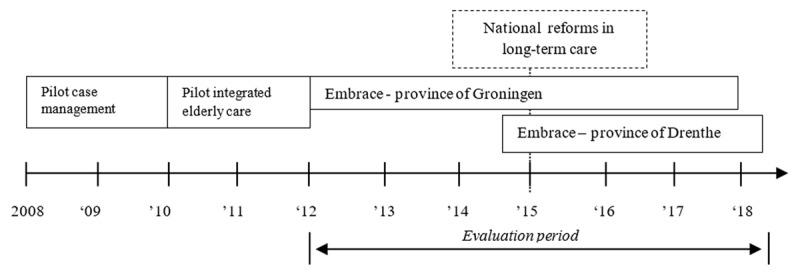
Timeline with the history of Embrace.

Embrace was preceded by two pilot studies on case management for older adults (2008–2011) and started as an integrated care service in 2012 in three municipalities in the eastern part of the province of Groningen. Embrace started in the province of Drenthe in 2014. The history of Embrace is shown in Figure 1. The national reforms in long-term care will be discussed in the paragraph on the Dutch healthcare system.

A multidisciplinary team from University Medical Centre Groningen initially developed the programme together with a health insurer and a health care provider. After the pilot studies, regional steering committees were installed in which multiple stakeholders participated, such as providers, payers and elderly representatives. The multidisciplinary team from University Medical Centre Groningen, among which were programme coordinators, advised and supported the steering committee and the local Elderly Care Teams. Furthermore, they monitored and evaluated the programme. Older adults and representatives’ organizations participated in regional Sounding Boards. Daily coordination of care was done by the Elderly Care Teams.

### Dutch Healthcare System

The Netherlands has a statutory health insurance system which is universal and mandatory [14]. Health insurers provide a package of covered benefits which, under the Health Insurance Act (ZVW), includes care provided by general practitioners (GP), hospitals, community nurses and some mental healthcare. Innovative health programmes or interventions that are not yet included in the benefit package covered by the healthcare insurers can apply for admission. These programmes can enter into temporary reimbursement agreement, conditional on studying clinical results and cost-effectiveness. If results prove positive, reimbursement may be continued as part of the general benefit package.

Primary care is provided by group practices, two-person practices or GPs working solo. In primary care, 75% of the payment is a mix of capitation and fee-for-service for core activities. There are also bundled payments for programmatic multidisciplinary care for chronic conditions and pay-for-performance contracts.

Not included under social health insurance are prevention and social support. These are funded by municipalities with funds from general taxation. This arrangement has been in place since the long-term care provision and financing reforms in 2015 [15,16] which aimed at more efficient coordination and procurement of services [16]. Health insurers became risk-bearing and responsible for the coordination and procurement of services in community nursing, primary care and hospital care under the Health Insurance Act (ZVW). Municipalities became responsible for social care and support under the Social Support Act (WMO), both for procurement and risk. Regional procurement offices were then commissioned to procure chronic institutional care and intensive home care, for which the central government bears the risk under the Long-Term Care Act (WLZ) [14].

These reforms were intended to keep health care and social support accessible and affordable in the long run. The aim was to enable older adults to live at home for as long as is possible and desirable, and to bring the responsibility and decision-making closer to citizens, i.e. in the municipalities. Informal care and the local community were intended to provide more support. However, the reforms led to problems in both coordination and procurement. Intensive home care, community nursing and social care and support now have different purchasers each with their own separate budgets and responsibilities that do not align. Great difficulties emerged in the coordination of services the anticipated improvements in efficiency were therefore not achieved. Moreover, perverse and non-aligned incentives led to unanticipated cost-shifting between the schemes [16].

### The Embrace payment model

Various funding sources, mostly grants, were used for the development, implementation and evaluation of Embrace. This was necessary, since elements of the intervention were not reimbursable at that time under various funding schemes (i.e. the Social Support Act, Health Insurance Act, Exceptional Medical Expenses Act and Long-Term Care Act).

Table 2 provides an overview of the way that the intervention was reimbursed since 2015 in the province of Groningen, i.e. the Elderly Care Teams, yearly screening, multi-disciplinary patient health records, self-management support and the prevention programme. The payment model was clearly fragmented over multiple payment schemes. Providers did not share risk or savings, neither was the payment related to health outcomes. Further details of the payment model, including that of Embrace in the province of Drenthe, are described in the Supplementary Materials, **Text 1**.

**Table 2:** The Embrace payment model in the province of Groningen (since 2015): who pays for what?

Programme elements	Reimbursed by	Covered by

Elderly Care Teams, yearly screening, multi-disciplinary patient health records, self-management support and prevention programme	Health insurer	Innovation reimbursement granted by Dutch Healthcare Authority (2014–2017)
*Case management for frail older adults* provided by social workers, self-management support and prevention programme and community care activities	Municipalities	Social Support Act (WMO)
*Case management for older adults with complex care needs* provided by district nurses and GP practices	Health insurer	Health Insurance Act (Zvw)
*Medical case management* provided by Elderly Care physicians and general practitioners	Regional procurement offices	Long-Term Care Act (WLZ)

## Impact

The outcomes of Embrace were evaluated in terms of the Triple Aim outcomes in multiple studies: a Randomised Controlled Trial (RCT), several pretest-posttest studies and qualitative studies. The RCT of 12 months of Embrace was done in the province of Groningen (2011–2013) [19]. In a longitudinal study in the province of Groningen (2012–2015), with 1.308 older adults included at the start, the outcomes were assessed in the intervention group at 12, 24 and 36 months and compared with the baseline measurement [21]. This study was repeated in the province of Drenthe (2015–2016) and the outcomes were assessed at 12 and 24 months, with 1.063 older adults included [22]. An overview of the different journal articles and reports on these studies is to be found in the Supplementary Materials Table 2. The main results per aim are summarized in the following paragraphs.

### Perceived quality of care

The Embrace intervention improved the quality of care as perceived by both older adults and professionals [24]. Also, professionals reported an increase in the level of implementation of integrated care, and case managers reported that they had a better overview of the status of the older adults [24]. Indeed, it proved possible to deliver more proactive and preventive measures and care [20]. Professionals experienced their new roles as satisfying, rewarding and challenging, but they also mentioned feelings of uncertainty and indicated that the high caseload led to stress and role conflicts [20].

### Health outcomes

After twelve months it was not possible to demonstrate any clear benefits for health, wellbeing and self-management for all the risk profiles, compared to the control group, in a Randomized Clinical Trial [25]. However, the long-term outcomes of Embrace were beneficial overall, particularly for older adults with complex care needs who received individual care. It seems that Embrace deferred the declining trends in general health and wellbeing associated with ageing in this group, compared to baseline [21,22,26]. The prevalence and severity of health-related problems in such areas as mental functioning, physical health and mobility did reveal long-lasting decreases after 12 months within the intervention-group of the RCT [27]. Collaborative goal planning helped the older adults in this intervention group to formulate and attain health related goals [28]. Furthermore, a qualitative study on the experiences of participating older adults with Embrace, showed Embrace met the health and social needs of older adults, reinforced their ability to stay in control and made them feel safe and secure [23].

### Costs

The cost-effectiveness study (RCT) revealed that Embrace was more effective, but also more expensive compared with the care-as-usual group in the first 12 months after implementation (n = 1.131) [29]. The longitudinal study in the province of Groningen showed that the costs of health and social care did not change over three years, compared to baseline, for older adults with complex care needs and for those who are frail [21]. In the case of the robust older adults, the healthcare costs increased (primary care, hospital care and dental care) in the third year [21]. The longitudinal study conducted in the province of Drenthe (2015–2016), showed some increase in the total costs of health and social care after 24 months, which was in line with the benchmark for older adults who did not participate in Embrace [22]. A summary of changes in costs per risk profile evaluated in the longitudinal studies is presented in Supplementary Materials **Table 3**.

## Why the programme ended

The summarized results would, at first glance, appear to render the programme futile; however, we now expand on the circumstances and issues that divert and absorb focus and effectiveness.

### Reforms and incentives

The ‘innovation reimbursement agreement’ of Embrace, granted by the Dutch Healthcare Authority for the Health Insurance Act, ended in July 2017. This agreement made it possible for Embrace to assess the long-term results, and if they were deemed positive, to have the intervention reimbursed on a permanent basis under the Health Insurance Act. The basis for this grant was a multi-stakeholder agreement, in which the intension and aim were embedded to scale up when Triple Aim outcomes were positive. One of the programme coordinators summarised what happened when the agreement ended: “The health insurer withdrew as a result from the evaluation of long-term outcomes. The main cause was that expected cost savings were not realised. For many stakeholders, this came by surprise. Municipalities were not planning on taking this over, financially. The largest health care provider had financial and organisational challenges itself.”

When the innovation reimbursement agreement ended, there was no alternative payment model. Embrace was halted in the province of Groningen in 2017 and in the province of Drenthe in 2018.

The reforms in long-term care (2015) had major consequences for the development of a payment model after the agreement ended in 2017. The reimbursement of health care and social support for older adults participating in Embrace were divided over two funding schemes (the Health Insurance Act and Social Support Act). Under these funding schemes, important elements of Embrace that were covered by the innovation reimbursement agreement until July 2017, could however not be reimbursed: the screening activities and risk stratification, the Electronic Elderly Record, and the self-management support and prevention programme. For municipalities and health insurers it was not appealing to invest in such preventive activities, as any potential cost savings would fall under another funding scheme (i.e. the Long-Term Care Act). Furthermore, the regional procurement offices were not risk-bearing for this scheme, and therefore not incentivised to invest and save costs [16,30].

### Fidelity to the protocol

One of the factors that influenced the outcomes of Embrace, was the lack of fidelity to the protocol. For example, not all older adults who had the risk profile ‘frail’ based on the risk assessment, received case management in accordance with the intervention protocol. In practise nearly half of these older adults were no longer receiving case management after a while and their risk profile was adjusted to ‘robust’ [21].

One of the reasons for the lack of fidelity might have been that the case managers caseloads in the Elderly Care Team increased since the start of Embrace. That is what the programme coordinators observed. The case managers were more or less urged to stop contacting the older adults if no imminent or urgent need for care or support was identified (or such needs ceased). Short term financial results of organizations outweighed long term impact, and daily issues required immediate attention. Or as one programme coordinator remarked: “during meetings we ask: are you only extinguishing fires or are you pro-actively and systematically screening the elderly?”.

### Shared values and understanding

In search for local implementation sites, program coordinators experienced that some GP practices did not fully share the Embrace vision and preferred more case-finding approach of including participants. One of the program coordinators described this challenge:

“When you try to implement and normalise in practise the [integrated care] vision that has a scientific foundation, minor derivations happen during the process. Then you think: they are taking the strength out of the intervention. The strategy is to stratify all older adults aged 75 years and older. One GP practice says, “let try that” and really is willing to do so. The other GP practise says, “I don’t believe in this approach”. That’s what you are navigating in between”.

Whether this lack of shared values influenced the fidelity to the protocol of participating GP practices, was not evaluated.

It was clear that the implementation of Embrace had impact on the management of the GP practise, the clinical information system, and required certain skills and capacity of staff. The intervention therefore had to fit the long-term vision and strategy of a GP practice and of the other local organizations in the primary, secondary and social care sector. This alignment is not always custom, as one of the programme coordinators remarked: “Implementing such a programme [Embrace], that is about managing a GP practise and about expertise, means examining where to connect. […]. Some GP’s say to me: why does it matter to me this region deals with a declining and ageing population. I have 1500 patients and together with my GP assistant, I will manage to sit it out. Why should I think about effective health care in this region?”.

### Legacy of Embrace

Although Embrace ultimately ended in 2018, there still are prospects for the revival of the programme. One of the Embrace programme coordinators has been asked by the Ministry of Health, Welfare and Sports to start developing a national framework for integrated people-centred health services for older adults. This framework will be based on the experiences of Embrace and similar programmes in the Netherlands and will be in line with the World Health Organization’s global strategy on integrated person-centred health services [2]. The national framework is intended to be used as a reference for policymakers, for drafting new legislation, for deciding on reimbursement policies and to guide payers and health and social service organizations in developing new integrated care programs. In preparation for the development of this framework, experts were consulted and multiple meetings were organized with all the relevant stakeholder groups, such as older adults, payers, health and social service organizations and research institutes.

The barriers that Embrace encountered in the quest for sustainable implementation were mainly at an organizational and policy level. The WHO strategic goals of *strengthening governance & accountability* and *creating an enabling environment* are therefore now explicitly included in the new national framework. In this way, future integrated care programmes for older adults should be better aligned and gain more support than Embrace had, to achieve the transformation as intended.

Furthermore, a revised integrated care programme for older adults started in 2017 in the Province of Drenthe, based on the building blocks of Embrace and on previous experiences of integrated care programmes in the region.

## Key lessons

### Incentivize the integration of care

From the start of Embrace, the Triple Aim outcomes were monitored and evaluated at the request of the health insurer. The long-term economic evaluation made it clear that the costs and benefits ended up accruing to different stakeholders and payers. Although the 2015 reforms aimed at more efficient coordination and procurement of services in long-term care, they produced new financial disincentives. From the perspective of individual organizations, it is not surprising that they acted in their own financial interests, as tends to happen in a healthcare system with managed competition [31]. We believe this stresses the importance of getting and keeping all the stakeholders aligned throughout the entire process until sustained implementation.

To create the *enabling environment* that the World Health Organization envisages [3], in our opinion, Embrace would need a novel integrated payment model that truly facilitates and incentivizes the coordination of health and social care services across different organizations and domains. The evidence base for innovative payment models that cover both health and social care is still limited [32]. Payment models for integrated care that show potential and are increasingly being used are population health management, bundled payments and shared savings, often with a pay-for-performance or pay-for-coordination element [32,33,34,35]. In the current Dutch long-term care system, a mechanism for sharing savings between the government, municipalities and insurers might also incentivise the coordination of health services as proposed by Alders & Schut (2019) [16]. The evaluations of nine Dutch population health management initiatives, however, show that the implementation, including the alternative payment models, turned out to be difficult, complex and time consuming [36]. Joining budgets and aligning the financial incentives with aims of the initiatives was one of the eight guiding principles that were identified.

One example in the Netherlands of a bundled payment model of this kind for an integrated care programme is to be found in the Care Chain Frail Elderly programme [37]. The intervention targets frail older adults and provides care coordination and case management. In this case the 2015 reforms are seen as an improvement, as they facilitate the bundled payment model which currently has limited scope including mainly primary care. Although the programme has the ambition to include more social care services and focus more on prevention, it faces challenges in cross-domain collaboration and in finding sustainable funding among the various Dutch funding schemes for health and social care.

### Prerequisites for change

During the years when Embrace was funded by grants or based on temporary financial agreements, most prerequisites seemed in place at the level of governance of the programme and communication [6]: participating organizations had joined up, the protocol was developed, the clinical information system was put in place, providers’ staff were trained. We think these conditions were necessary to enable the shift from providing reactive and problem-oriented healthcare to proactive and preventive health services. However, Embrace was challenged at the micro-level of providers by a combination of lack of fidelity (i.e. whether the intervention was delivered as intended) and consequently insufficient reach (i.e. whether the target population received the intervention) [38]. It shows, in our opinion, that providers’ staff need to share the values and understand the aims of the intervention [6] and even with extensive training and support it appears a challenge to make a change at managerial and clinical level. Staff have to experience providing proactive and preventive support as an improvement compared to providing care as usual and it should lead to higher work satisfaction, in order to stay engaged. If we recognize improving the *experience of providing care* as an important aim of the programme, and monitor it, the Triple Aim would need to be expanded to a Quadruple Aim [39].

### Future actions and research

The key lessons from Embrace are clearly not limited to the (Northern) Netherlands and are worth sharing internationally. In our opinion, multiple frameworks and methods would be useful to provide a better understanding of the complexity of the sustained implementation of current and future integrated care services. First, prior to large-scale implementation, an evaluation framework such as the Consolidated Framework for Implementation Research (CFIR) could help to identify critical barriers, as used by Warner et al. (2018) for an online frailty tool [40]. Secondly, to obtain a deeper understanding of why some elements work in particular contexts, i.e. the mechanisms, process evaluation is crucial in addition to evaluating the outcomes. Using a realist evaluation method for this could create a better understanding of how the context, the mechanisms and the outcomes interact [41,42,43,44]. This method is suitable for evaluating complex social interventions that are theory-based, but it is also flexible and accounts for the realities in which interventions are implemented [42]. Thirdly, Normalization Process Theory (NPT) tools could be used to develop, evaluate and also implement future complex interventions in clinical practise [45]. These tools are suitable for assessing factors that clinical staff perceive as relevant when implementing a new intervention, and to which the implementation plan can then be tailored. Finally, collective case studies could contribute to the limited evidence base. Ideally these use a comprehensive framework that enables the comparison of such health services in different contexts, as proposed by González-Ortiz et al. (2018) [7] and as was done in the European projects SELFIE [46] and INTEGRATE [47].

### Limitations

Although we have collected and analysed data on Embrace in multiple ways, the semi-structured interviews were limited to programme coordinators. This might have led to an over-emphasis on the main barriers from their perspectives.

One of the factors that might have influenced the effectiveness, yet not included in this paper, is the interoperability of the Elderly Care Record with the IT systems of health care providers. To this date, within the Dutch primary care sector such interoperability issues occur, however we did not assess these issues or identified them in the data analysed.

## Conclusions

This retrospective assessment of Embrace (2012–2018) underscores the importance of an enabling context, including a payment model that truly incentivizes the integration of health and social care services. These prerequisites are crucial to transforming health services at an organizational and clinical level, from being reactive and problem-solving to becoming proactive and preventive. Without the right incentives, monitoring, training and support, and shared values and understanding, this transformation will not last. However, with a new national framework for integrated person-centred health services for older adults in sight, there are still prospects for revival of Embrace.

## Additional Files

The additional files for this article can be found as follows:

10.5334/ijic.5434.s1 Supplementary Text 1. Details of the Embrace payment model.

10.5334/ijic.5434.s2 Supplementary Table 1. Details of the Embrace payment model.

10.5334/ijic.5434.s3 Supplementary Table 2. Overview of publications on Embrace included in this study.

10.5334/ijic.5434.s4 Supplementary Table 3. Change in costs per risk profile*, compared to baseline.
