# Supplementary material for: Integrated Care for Older Adults: A Struggle for Sustained Implementation in Northern Netherlands
Source: Int J Integr Care. 2020 Jul 13;20(3):1. doi: 10.5334/ijic.5434 (PMC7366864; doi:10.5334/ijic.5434)
Supplement: Supplementary Text 1. — Details of the Embrace payment model. [file ijic-20-3-5434-s1.pdf]

Supplementary Materials
Supplementary Text 1. Details of the Embrace payment model

Embrace started in the province of Groningen in 2012. The project costs for developing the intervention were funded by the National Care for the Elderly Programme (NPO) which is coordinated by The Netherlands Organization for Health Research and Development (ZonMw). The costs of Elderly Care Teams (i.e. the intervention costs) were funded by the Dutch Healthcare Authority (NZa) for six months, followed by the NPO for six months and the Health Insurer Fund for innovative projects (SAG) since these new roles were not reimbursable at that time under the various funding schemes (i.e. the Social Support Act, Health Insurance Act and Long-Term Care Act). From July 2014 to July 2017 the intervention was reimbursed by the health insurer Menzis, based on an agreement for innovative programmes granted by the Dutch Healthcare Authority (NZa). The costs of the project coordination were funded by reallocating the surplus on tariffs for the Elderly Care Teams.

Embrace started in the province of Drenthe in 2014. The intervention was first funded by using a reimbursement code granted by the Dutch Healthcare Authority for the National Care for the Elderly Programme (NZa beleidsregel NPO) (2014), followed by a grant from the National Care for the Elderly Program run by The Netherlands Healthcare Organization for Health Research and Development (ZonMw grant NPO projects), and finally by the health insurer Zilveren Kruis and the municipality of Emmen.
