# Supplementary material for: Integrated Care for Older Adults: A Struggle for Sustained Implementation in Northern Netherlands
Source: Int J Integr Care. 2020 Jul 13;20(3):1. doi: 10.5334/ijic.5434 (PMC7366864; doi:10.5334/ijic.5434)
Supplement: Supplementary Table 1. — Details of the Embrace payment model. [file ijic-20-3-5434-s2.pdf]

Supplementary Table 1. Characteristics of the intervention for each risk profile

	**Robust**	**Frail**	**Complex Care Needs**
Level of care and support	Low intensity	High intensity	High intensity
Coordination of care and support	Elderly Care Team	Elderly Care Team, case manager	Elderly Care Team, case manager
Contact	Unplanned: initiated by older adult or Elderly Care Team	Planned: once a month (on average)	Planned: twice a month (on average)
Length of individual care and support	Not applicable	6-12 months	6-12 months
Approach	Group	Individual and group	Individual and group
Focus	Self-management	Psychosocial	Health
