# Supplementary material for: Integrated Care for Older Adults: A Struggle for Sustained Implementation in Northern Netherlands
Source: Int J Integr Care. 2020 Jul 13;20(3):1. doi: 10.5334/ijic.5434 (PMC7366864; doi:10.5334/ijic.5434)
Supplement: Supplementary Table 2. — Overview of publications on Embrace included in this study. [file ijic-20-3-5434-s3.pdf]

Supplementary Table 2. Overview of publications on Embrace included in this study

Authors	Title	Region	Timeframe	Conclusions
Spoorenberg SLW, Uittenbroek RJ, Middel B, Kremer BPH, Reijneveld SA, Wynia K (2013) [19]	Embrace, a model for integrated elderly care: study protocol of a randomized controlled trial on the effectiveness regarding patient outcomes, service use, costs, and quality of care	Groningen	Not applicable	Description of the study protocol
Spoorenberg SLW, Wynia K, Fokkens AS, Slotman K, Kremer HPH, Reijneveld SA(2015)[23]	Experiences of Community-Living Older Adults Receiving Integrated Care Based on the Chronic Care Model: A Qualitative Study	Groningen	2011-2013 (12 month follow up)	Integrated care and support provided through Embrace met the health and social needs of older adults, who were coping with the consequences of aging
Spoorenberg, Uittenbroek, Wynia, Kremer, & Reijneveld, 2016 [21]	Uitkomsten van SamenOud: voor ouderen, kwaliteit van zorg, zorggebruik en kosten na 12, 24 en 36 maanden [Results of Embrace: for elderly, quality of care, care utilization and cost after 12, 24 and 36 months]	Groningen	2012-2015	Overall, the results of Embrace, quality of care, care utilization and long-term costs are mostly positive, especially for the elderly with the risk profile of complex care needs.
Soeters & Verhoeks, 2016 [30]	Analyse belemmeringen structurele bekostiging vier NPO projecten [Analysis of barriers for structural payment models of four NPO-projects]	Groningen, Drenthe	2009-2016	It is technically possible to finance Embrace within the current health system.
Uittenbroek, Kremer, Spoorenberg, Reijneveld, & Wynia, 2017 [24]	Integrated Care for Older Adults Improves Perceived Quality of Care: Results of a Randomized Controlled Trial of Embrace	Groningen	2011-2013 (12 month follow up)	Embrace improved the quality of care as perceived by older adults and participating professionals
Spoorenberg, Wynia, Uittenbroek, Kremer, & Reijneveld, 2018 [25]	Effects of a population-based, person-centred and integrated care service on health, wellbeing and self-management of community-living older adults: A randomized controlled trial on Embrace	Groningen	2011-2013 (12 month follow up)	This study found no clear benefits to receiving person-centred and integrated care for twelve months for the domains of health, wellbeing and self-management in community-living older adults
Uittenbroek, van Asselt, Spoorenberg, Kremer, Wynia , Reijneveld (2018) [29]	Integrated and Person-Centered Care for Community-Living Older Adults: A Cost-Effectiveness Study	Groningen	2011-2013 (12month follow up)	According to current standards, Embrace is not considered cost effective after 12 months. However, it could be considered worthwhile in terms of "risk profile improvements" for older adults with "Complex care needs," if society is willing to invest substantially.
Spoorenberg, Wynia, van Asselt, Jager, Kremer, Reijneveld., 2018 [22]	SamenOud in Zuidoost-Drenthe; Uitkomsten voor de ouderen, kwaliteit van zorg, zorggebruik en kosten na 12 en 24 maanden [Embrace in SouthEast-Dretnhe; Outcomes for elderly, quality of care, care utilization and cost after 12 and 24 months]	Drenthe	2015-2016	Overall, the results of Embrace, quality of care, care utilization and long-term costs are mostly positive, especially for the elderly with the risk profile of complex care needs.
Rietkerk, Uittenbroek, Gerritsen, Slaets, Zuidema & Wynia [28]	Goal planning in person-centred care supports older adults receiving case management to attain their health-related goals	Groningen	2011-2013 (12 month follow up)	Goal plans of Embrace participants aim at improving health-related problems concerning physical health, mobility, or support. Goals related to physical health are the most likely to be attained, while goals for mobility and pain are the least likely to be attained.
Wynia K, Uittenbroek RJ, van der Mei SF, Slotman K, Reijneveld (2018) [20]	Experiences of case managers in providing person-centered and integrated care based on the Chronic Care Model: A qualitative study on embrace	Groningen	2013	"Case managers found their new roles satisfying and challenging, although stressful at times. They felt they could make the difference. Ongoing training and support were found to be a prerequisite in helping to shift the focus towards person-centered and integrated care."
Wynia K, Spoorenberg SLW, Uittenbroek RJ, Jager M, Kremer HPH, Reijneveld SA (2018)[26]	Long-term advantages of person-centred and integrated care: results of a longitudinal study on Embrace. (poster abstract)	Groningen	2012-2015	"Overall, long-term outcomes of Embrace for the older adults are beneficial, particularly for older adults with complex care needs. It seems that Embrace has halted the declining trends in general health and well-being associated with ageing, as well as the related costs increase."
Spoorenberg SL, Reijneveld SA, Uittenbroek RJ, Kremer HP, Wynia K (2019)[27]	Health-Related Problems and Changes After 1 Year as Assessed With the Geriatric ICF Core Set (GeriatrICS) in Community-Living Older Adults Who Are Frail Receiving Person-Centered and Integrated Care From Embrace.	Groningen	2012-2013	"Embrace offers a route to counteracting the decline in physical, cognitive and social functioning associated with aging. Prevalence and severity of health-related problems for those with a problem at baseline decreased after 12 months".
