# Supplementary material for: Integrated Care for Older Adults: A Struggle for Sustained Implementation in Northern Netherlands
Source: Int J Integr Care. 2020 Jul 13;20(3):1. doi: 10.5334/ijic.5434 (PMC7366864; doi:10.5334/ijic.5434)
Supplement: Supplementary Table 3. — Change in costs per risk profile*, compared to baseline. [file ijic-20-3-5434-s4.pdf]

Supplementary Table 3. Change in costs per risk profile*, compared to baseline

		**Baseline** Average	**12 months after baseline** difference	**24 months after baseline** difference	**36 months after baseline** difference
Groningen study [19]	Robust	€ 4.957	576	720	2.549
	Frail	€ 9.091	2.499	3.357	-946
	Complex Care Needs	€ 17.725	-1.089	-1.489	-144
	Total group	*€ 10.435*	*662*	*870*	*486*
Drenthe study [20]**	Robust	€ 4.258	1.703	3.209	
	Frail	€ 8.832	2.379	4.627	
	Complex Care Needs	€ 18.280	761	2.204	
	Total	€ 8.957	1.610	3.313	

* Risk profile based on practise, not based on scores of the assessment.

** Costs of home care nursing and social support changed due to the long-term care reforms. These were not included in the baseline and introduced in the first 12 months.
